# Identification pyroptosis-related gene signature to predict prognosis and associated regulation axis in colon cancer

**DOI:** 10.3389/fphar.2022.1004425

**Published:** 2022-09-27

**Authors:** Kexun Zhou, Xuyu Gu, Huaicheng Tan, Ting Yu, Chunhua Liu, Zhenyu Ding, Jiyan Liu, Huashan Shi

**Affiliations:** ^1^ Department of Radiotherapy, Cancer Center, West China Hospital, Sichuan University, China; ^2^ School of Medicine, Southeast University, Nanjing, China; ^3^ Department of Pathology and Laboratory of Pathology, State Key Laboratory of Biotherapy, West China Hospital, West China School of Medicine, Sichuan University, China; ^4^ Department of Radiotherapy, Cancer Center and State Key Laboratory of Biotherapy, West China Hospital, Sichuan University, China

**Keywords:** pyroptosis, colon cancer, genes signature, prognosis, immunotherapy

## Abstract

**Background:** Pyroptosis is an important component of the tumor microenvironment and associated with the occurrence and progression of cancer. As the expression of pyroptosis-related genes and its impact on the prognosis of colon cancer (CC) remains unclear, we constructed and validated a pyroptosis-related genes signature to predict the prognosis of patients with CC.

**Methods:** Microarray datasets and the follow-up clinical information of CC patients were obtained from the Gene Expression Omnibus (GEO) and the Cancer Genome Atlas (TCGA) databases. Candidate genes were screened out for further analysis. Various methods were combined to construct a robust pyroptosis-related genes signature for predicting the prognosis of patients with CC. Based on the gene signature and clinical features, a decision tree and nomogram were developed to improve risk stratification and quantify risk assessment for individual patients.

**Results:** The pyroptosis-related genes signature successfully discriminated CC patients with high-risk in the training cohorts. The prognostic value of this signature was further confirmed in independent validation cohort. Multivariable Cox regression and stratified survival analysis revealed this signature was an independent prognostic factor for CC patients. The decision tree identified risk subgroups powerfully, and the nomogram incorporating the gene signature and clinical risk factors performed well in the calibration plots.

**Conclusion:** Pyroptosis-related genes signature was an independent prognostic factor, and can be used to predict the prognosis of patients with CC.

## Introduction

Worldwide, colon cancer (CC) ranks the fifth most frequent cancer and leading cause of cancer death, as nearly 1.10 million newly diagnosed cases and 0.58 million deaths reported in 2020 (Sung et al., 2021). With the development of precision medicine, significant efforts have been made to refine the personalized management of CC. Generally, surgery, neoadjuvant and adjuvant treatments are the cornerstone in the treatment of early-stage CC. For advanced CC, various active drugs were involved, which included multidrug-chemotherapy regimens, targeted therapies (e.g., Bevacizumab, Cetuximab, Regorafenib), and immunotherapies. Besides, strategies for individual patients are mainly based on prognostic factors which have been verified in previous studies, such as the ratio of positive lymph node, the status of microsatellite instability (MSI) (Ribic et al., 2003; Sargent et al., 2010; Sabbagh et al., 2014; Gill et al., 2015). However, the value of existing prognostic markers is not sufficient. For example, it is well established that patients with stage III CC could benefit from the adjuvant therapy of fluoropyrimidines alone, as the risk of death decreases 10–15% (Twelves et al., 2005). But for stage II disease, the adjuvant chemotherapy does not improve survival by more than 5%. (Quasar Collaborative Group et al., 2007). In addition, the predictive value of microsatellite instability (MSI) remains controversial. A retrospective study conducted by Sargent et al. indicated that in stage II CC with defective DNA mismatch repair (dMMR), adjuvant therapy was associated with reduced overall survival (Sargent et al., 2010). In contrast, by analyzing data from QUASAR study, Hutchins G et al. found, that dMMR could not predict benefit or detrimental impact of chemotherapy (Hutchins et al., 2011). This finding has been borne out in another study as well (Bertagnolli et al., 2011). Therefore, discovery of new biomarkers is required to discriminate high-risk subsets who most likely benefit from treatment and avoid unnecessary toxicities.

Pyroptosis in the form of a novel cell death, plays an important role in the development of cancer. As Kolb R et al. summarized the role of various inflammasome factors in cancer progression and therapy, the key components of pyroptosis, such as gasdermin (GSDM) proteins, were identified associated with tumorigenesis, invasion, and metastasis (Kolb et al., 2014). Meanwhile, the impact of the GSDM on the occurrence and prognosis of lung adenocarcinoma (LUAD) was evaluated by Wei J et al. The results indicated that GSDMC is significantly upregulated in LUAD tissues and the overexpression of GSDMC was an independently negative prognostic factor in patients with LUAD (Wei et al., 2020). In addition, pyroptosis could also active and promote the release of inflammatory cytokines, leading to the epithelial-to-mesenchymal transition (EMT), cancer invasion and migration (Tulotta et al., 2019; Fang et al., 2020).

Based on these findings, significant efforts have been made to identify the implication of pyroptosis in various cancer types. And existing evidence verified that pyroptosis-related genes could be served as independent prognostic factors in some solid tumors, such as hepatocellular carcinoma (HCC), gastric cancer (GC), and ovarian cancer (OC) (Shao et al., 2021; Ye et al., 2021; Zheng et al., 2021). For example, Zheng S et al. developed five pyroptosis-related gene signature to analyze its survival prediction value in HCC. The results suggested the signature could well predict the outcomes of HCC patients (Zheng et al., 2021). These findings have been borne out in other studies, as pyroptosis-related genes were identified as an important role to predict adverse prognosis and guide treatment in GC and OC patients (Shao et al., 2021; Ye et al., 2021)**.**

Furthermore, with the growing field of immune oncology, increasing attention has been focused on the role of pyroptosis-related gene signature in immunotherapy response. Wang Q et al. demonstrated, that inflammation triggered by pyroptosis could synergize the efficacy of anti-PD-1 therapy (Wang et al., 2020a). However, its functional impact in CC remained a critical knowledge gap. Thus, we performed a systematic study to explore the pyroptosis-related genes and their prognostic value for patients with CC. The association between the gene signature and immune microenvironment was also validated.

## Methods

### Dataset

The methods have been well-established in previous studies (Irizarry et al., 2003; Sun et al., 2020). Briefly, a total of 1086 CC patients were included in our study across different platforms. The microarray dataset GSE14333 downloaded from Gene Expression Omnibus (GEO) was used as train set, while GSE226, GSE17536, GSE177, GSE41258, and GSE250 were integrated into a new validation cohort. Meanwhile, the RNA-seq data of 432 CC patients with corresponding clinical information was downloaded from The Cancer Genome Atlas (TCGA), as another validation set. The sva package (COMBAT) was used to remove the batch effects, while the robust multichip average (RMA) algorithm was performed to normalize the raw CEL files. All microarray and RNA-seq data were log2 transformed.

### Development and validation of the pyroptosis-related genes prognostic model

Thirty-three pyroptosis-related genes were extracted from previously published studies and presented in Supplementary Table S1 (Barbie et al., 2009; Liberzon et al., 2011; Wang et al., 2018a; Tang et al., 2020; Yu et al., 2021). Based on these genes, a single-sample gene set enrichment analysis (ssGSEA) was used to construct the pyroptosis-related risk score (PRS). To evaluate the significance of different cancer hallmarks in CC, univariate Cox proportional-hazards (Cox-PH) regression model was performed, which was based on the R package ‘survival’. The package of ‘wgcna’ (weighted gene co-expression network analysis) was performed to identify the module which was most correlated with pyroptosis based on transcriptome profiling data and ssGSEA scores. Subsequently, a least absolute shrinkage and selection operator (LASSO) Cox regression model was utilized to narrow down the candidate genes, screening out the most robust prognostic markers. The PRS was established as follows, and the Z-score method was used to normalize ssGSEA scores and PRS when necessary:

$$PRS=\sum iCoefficient\left({mRNA}_{i}\right)\times Expression\left({mRNA}_{i}\right)$$

Patients with CC from GEO and TCGA were divided into low- and high-risk according to the median risk score. Kaplan-Meier analysis was employed to compare the OS between the two groups. With the R package ‘survival ROC’, time-dependent receiver operating characteristic (tROC) analysis was performed, and the areas under the curve at different time points [AUC(t)] of all the variables were compared. To evaluate the prognostic value in the pooled cohort, meta-analysis (I^2^ > 30%, random-effects model) was conducted. And non-negative matrix factorization (NMF) consensus clustering was used to divide one cohort without a full-scale gene signature expression pattern into different clusters according to the best k value with the R package ‘nmf’.

### Independent prognostic analysis of PRS

Clinical information of CC patients was extracted from the GEO and TCGA (Supplementary Table S2), then was analyzed in the regression model, combined with PRS. Univariate and multivariable Cox regression models were employed for the analysis. A decision tree for risk stratification with the R package ‘rpart’ was constructed, using recursive partitioning analysis (RPA). A nomogram and a calibration curve were plotted using the R package ‘rms’.

### Functional enrichment analysis

The GSEA analysis was also performed to identify the differential Gene ontology (GO) and Kyoto Encyclopedia of Genes and Genomes (KEGG) pathways, as well as the relevance between the PRS and immune cells.

### Drug sensitivity

Spearman correlation analysis was performed to analysis the relationship between the prognostic pyroptosis-related genes and drug sensitivity.

## Results

### Pyroptosis is the risk factor for overall survival in CC patients

**FIGURE 1:**
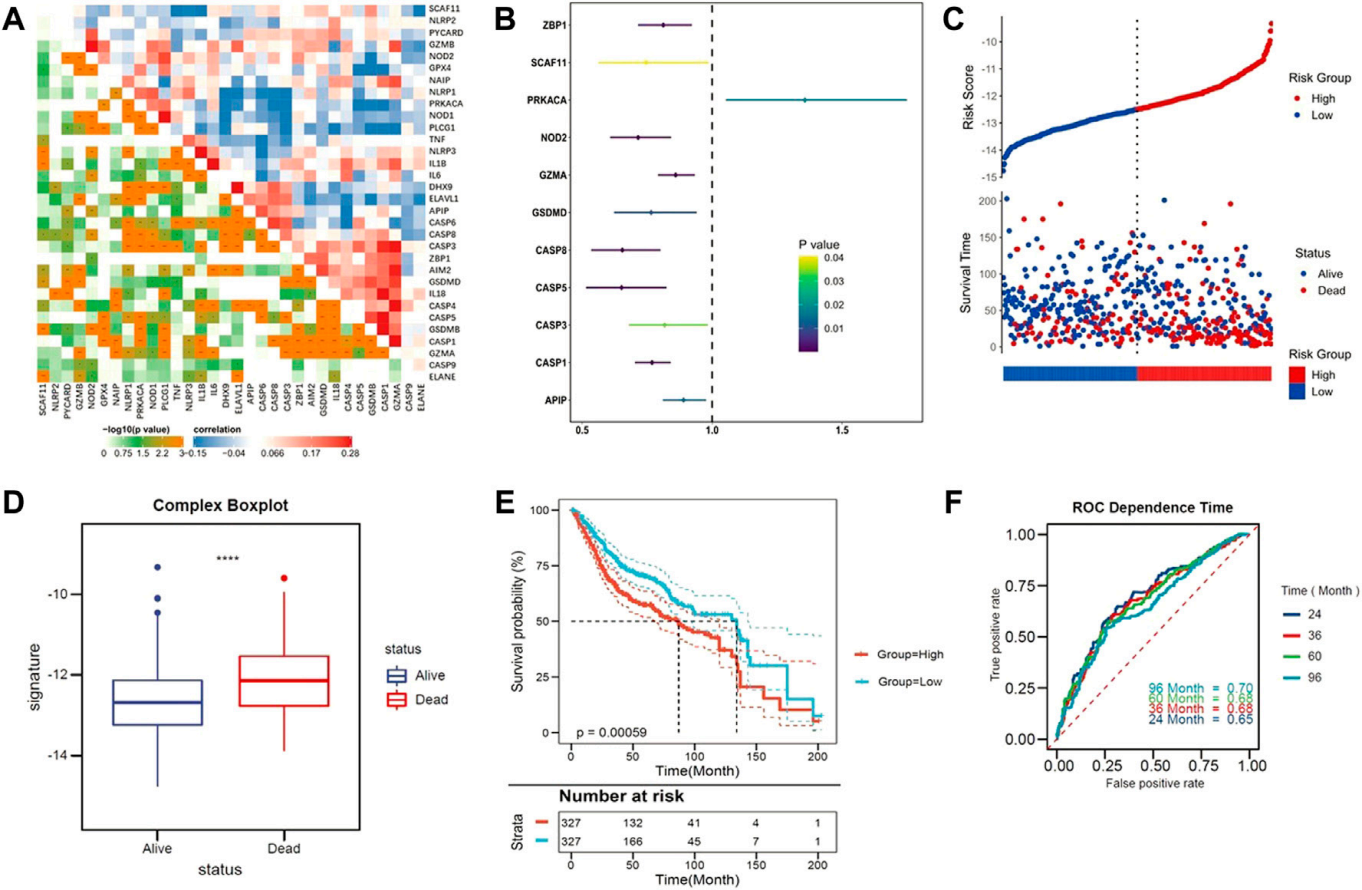
Identify the pyroptosis-related genes. **(A)** The correlation heatmap containing the candidate pyroptosis-related genes. (Red: positive correlation; Blue: negative correlation. The strength of the relevance was represented by the depth of the color). **(B)** Univariate cox regression analysis of OS for each pyroptosis-related genes. **(C)** Patients with low-risk scores had a survival advantage over patients with high-risk scores. **(D)** Pyroptosis ssGSEA scores were significantly elevated in patients who died during follow up. **(E)** Kaplan–Meier curves for the OS of patients in the high- and low-risk groups. **(F)** ROC curves indicated the predictive efficiency of the risk score.

First, we identified that pyroptosis was an independent risk factor among various cancer hallmarks for patients with CC. The correlation between the candidate pyroptosis-related genes was presented in Figure 1A. Survival-related genes were screened out by univariate Cox regression analysis. With the criteria of *p* < 0.05, eleven genes stood out (ZBP1, SCAF11, PRKACA, NOD2, GZMA, GSDMD, CASP8, CASP5, CASP3, CASP1 and APIP), and were identified as low-risk factors (Hazard ratio (HR) < 1), except for PRKACA (HR > 1) (Figure 1B).

Based on the Z-scores of the pyroptosis ssGSEA score, 654 CC patients were divided into low- and high-risk subgroups equally. Patients with low-risk scores had a survival advantage over that with high-risk scores Figures 1C, D shows that Z-scores of the pyroptosis ssGSEA were significantly elevated in dead patients compared with patients alive during the follow-up. Statistical difference in overall survival (OS) was observed between two subgroups (*p* < 0.0001, Figure 1E). As tROC demonstrated, the area under the ROC curve (AUC) was 0.70 for 96-months, 0.68 for 60-months, 0.68 for 36-months and 0.65 for 24-months survival (Figure 1F).

### Establishment of pyroptosis-related genes signature for prognosis

**FIGURE 2:**
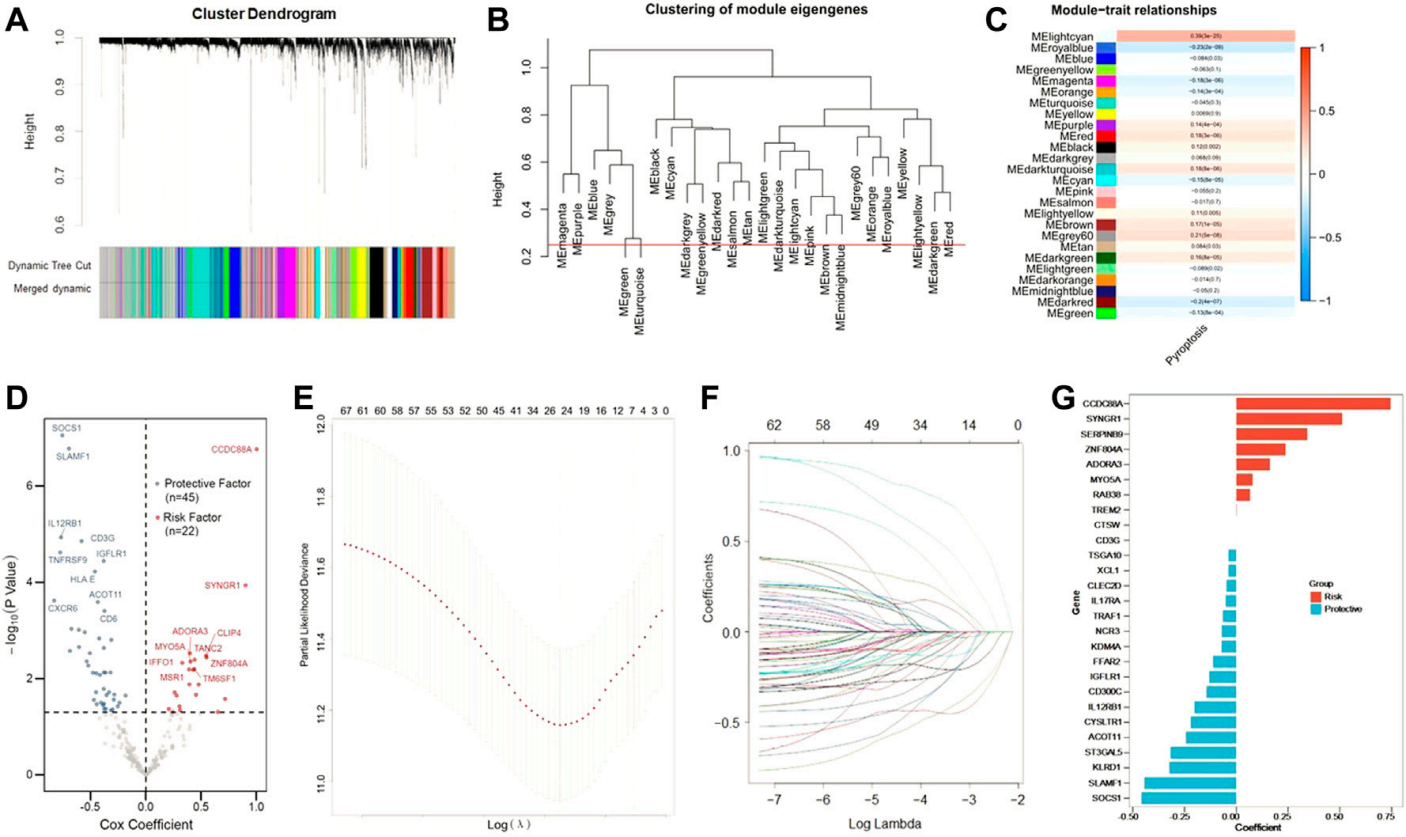
Construction of risk signature in the training cohort. **(A)** WGCNA was performed with whole-transcriptome profiling data and pyroptosis ssGSEA Z-score. **(B)** A total of 26 non-grey modules were identified after merging. **(C)** The red module depicting the highest correlation (r = 0.18, p = 3e-06) was considered the most correlated with pyroptosis. **(D)** Sixty-seven promising candidates were identified among hub genes extracted from the red module. **(E,F)** The LASSO Cox regression model was used to identify the most robust markers, with an optimal λ value of 0.0617. **(G)** Distribution of LASSO coefficients of the pyroptosis-related gene signature.

Next, we tried to screen out the promising candidates, in order to establish a robust pytoptosis-related gene signature to predict the survival of CC patients. Using whole-transcriptome profiling data and pyroptosis ssGSEA Z-scores in the training set, WGCNA was developed. With an optimal threshold power of *β* = 5 (Supplementary Figure S1), 26 non-grey modules were generated (Figure 2A). The module which was considered the most correlated with pyroptosis was represented by lightcran (*r* = 0.39, *p* = 3e-25) (Figures 2B,C). Hub genes extracted from the lightcran module were used for further univariate Cox regression analysis, with a threshold of *p* value for GS set as <0.0001. With a threshold of *p* value for uni-Cox of <0.05, 67 potential candidates were identified (Figure 2D). Among of them, 45 were protective markers, while 22 were risk markers. Furthermore, the most robust markers for prognosis were identified by the LASSO Cox regression model. To address the over-fitting, ten-fold cross-validation was applied, with selected optimal λ value of 0.0249 (Figures 2E,F). After validation, with individual nonzero LASSO coefficients, CCDC88A, SYNGR1, SEPRINB9, ZAF804A, ADORA3, MYO5A, RAB38, TREM2, CTSW, CD3G, TSGA10, XCL1, CLEC2D, IL17RA, TRAF1, NCR3, KDM4A, FFAR2, IGFLR1, CD300C, IL12RB1, CYSLTR1, ACOT11, ST3GAL5, KLRD1, SLAMF1 and SOCS1 remained. The distribution of LASSO coefficients of the gene signature was presented in Figure 2G. As a result, the PRS formula was developed as follows: $PRS=\sum iCoefficient\left({mRNA}_{i}\right)\times Expression\left({mRNA}_{i}\right)$ . The expression level of each gene was log2 normalized.

### PRS is an accurate predictor for overall survival of CC patients

**FIGURE 3:**
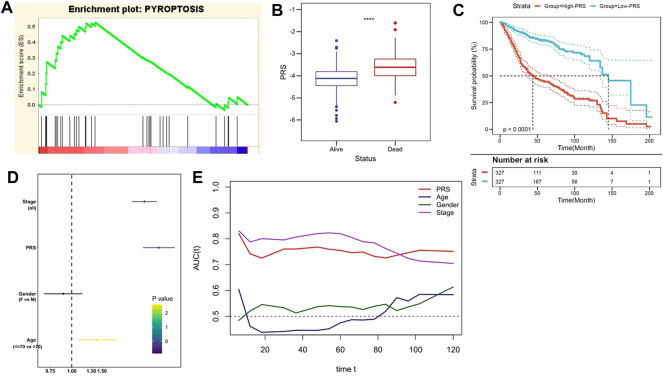
The gene signature predicts worse survival in the training set. **(A)** GSEA confirmed the status of pyroptosis in the two subgroups. **(B)** The follow up data indicated that PRS significantly decreased in patients alive. **(C)** Kaplan–Meier survival curve showed that patients with lower PRS enjoyed better outcomes. **(D)** Multivariate Cox regression analysis demonstrated that PRS was an independent risk factor for OS. **(E)** tROC analysis suggested that PRS was an accurate variable for predicting the survival.

Subsequently, the prognostic value of PRS was evaluated in the training and independent validation cohorts. In the training set, risk markers positively correlated with pyroptosis accumulated more in the high-PRS group, compared with the low-PRS group (Figure 3A). Furthermore, the follow-up data indicated that the risk score significantly decreased in patients alive (Figure 3B). As Kaplan–Meier survival curve indicated, patients with lower PRS enjoyed a statistically significant survival benefit than its competitor (*p* < 0.0001, Figure 3C). Multivariate Cox regression model involved various clinicopathological factors was performed, and the results revealed that TNM stage and PRS were two independent risk factors for OS (Figure 3D). In addition, tROC analysis suggested that PRS was an accurate variable to predict the survival of CC patients (Figure 3E).

**FIGURE 4:**
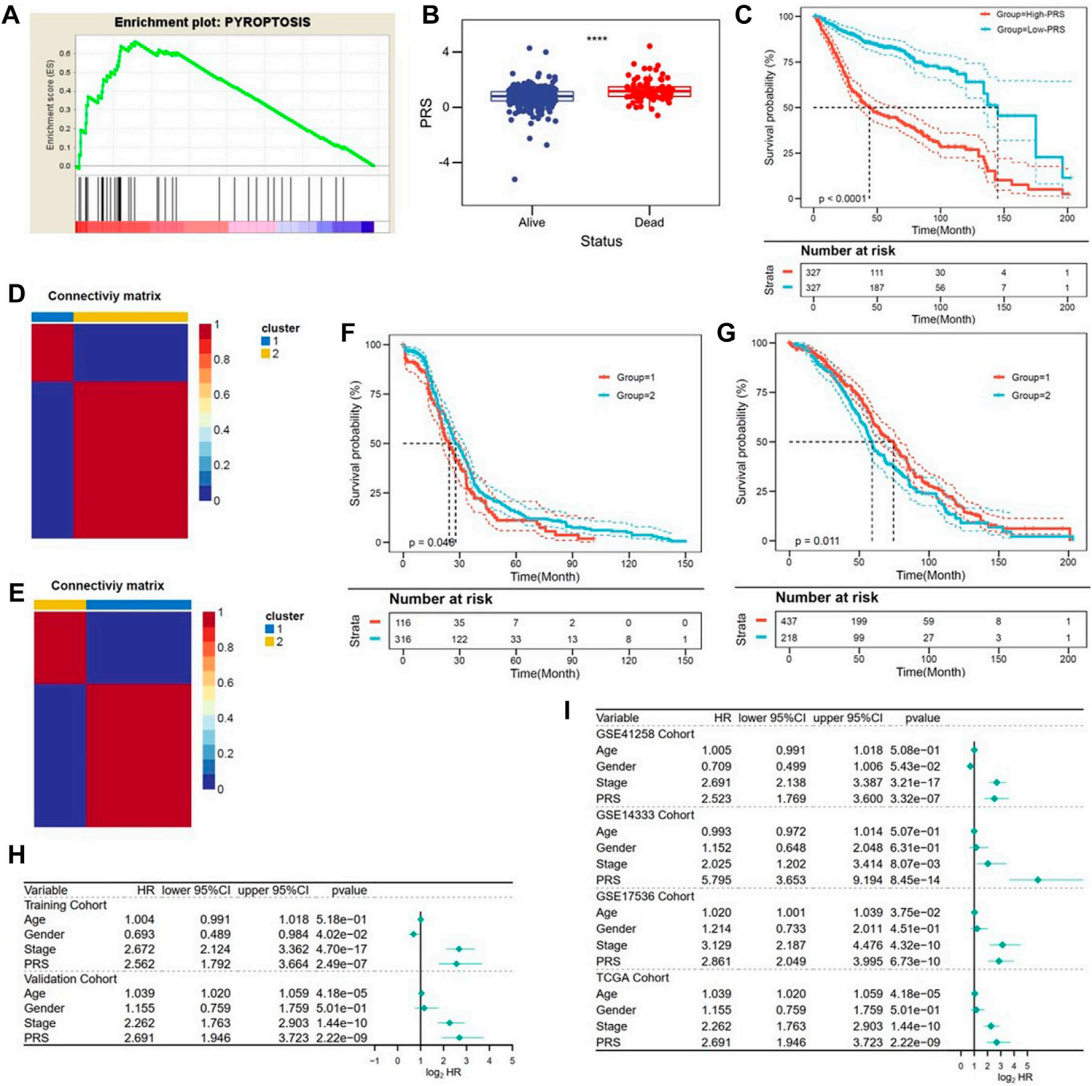
Validation of the gene signature in different series. **(A)** TCGA confirmed the pyroptosis status in the validation cohort. **(B)** PRS was significantly decreased in patients alive in the validation cohort. **(C)** Patients with lower PRS exhibited better prognosis in the validation cohort. **(D,E)** The best k value was chosen for NMF consensus clustering in the TCGA (Figure 4D) and GEO (Figure 4E) cohorts. **(F,G)** Statistical difference in OS was observed in NMF-derived clusters based on the expression pattern of the gene signature (TCGA: Figure 4F; GEO: Figure 4G). **(H,I)** Multivariate Cox regression analysis indicated that PRS was an independent risk factor for OS in the training and validation cohorts (Figure 4H), as well as in the all cohorts (Figure 4I).

The robustness of predictive value of the pyroptosis-related genes signature was also validated in other independent cohorts. Briefly, higher enrichment score of pyroptosis gene set was confirmed in the high-PRS group in the validation I cohort (Figure 4A). Also, patients alive had lower PRS, compared with the deceased (*p* < 0.0001, Figure 4B). Kaplan-Meier analysis confirmed the survival benefit in patients with lower PRS in the validation cohort (*p* < 0.0001, Figure 4C). Furthermore, NMF consensus clustering was used to divide both TCGA and GEO cohort into two groups (Figures 4D,E). The division was based on the best k value, which was the remaining expression pattern of the gene signature. The results revealed statistical difference in OS between NMF-derived groups (Figures 4F,G). Moreover, multivariate Cox regression analysis conquered that PRS was an independent risk factor for OS, not only in the training and validation cohort (Figure 4H), but also in the all cohorts (Figure 4I).

### PRS acts as an indicator of worse prognosis in the pooled cohort and a promising marker of therapeutic resistance

**FIGURE 5:**
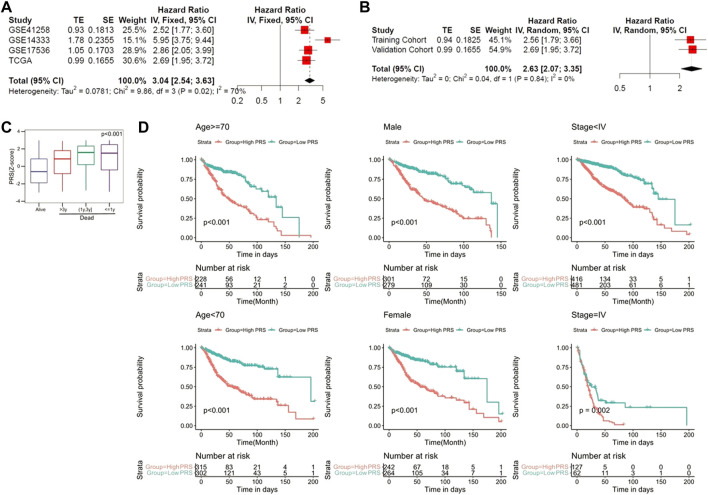
The gene signature serves as a valuable marker for poor survival in the pooled cohort. **(A)** Meta-analysis in the training and validation cohorts. **(B)** Meta-analysis for subgroup analysis. **(C)** PRS Z-scores were significantly elevated in deceased patients. **(D)** PRS discriminated high-risk patients in different clinicopathological including gender, age, and p-stage.

To evaluate the prognostic value of the pyroptosis-related genes signature in the pooled cohort including the training and validation cohorts, meta-analysis was performed. The results suggested that CC patients with higher PRS had worse prognosis than those with lower PRS (pooled Hazard ratio (HR) = 2.63, 95% CI 2.07–3.35) (Figures 5A,B). Subsequently, 1,096 patients were extracted for further investigation. PRS Z-scores were significantly elevated in those patients who died during the follow-up, especially in patients with shorter survival (Figure 5C). When the candidate patients were divided into virous subgroups, based on different clinicopathological features (age, sex and stage), PRS also helped to screen out high-risk patients with poor prognosis (Figure 5D).

**FIGURE 6:**
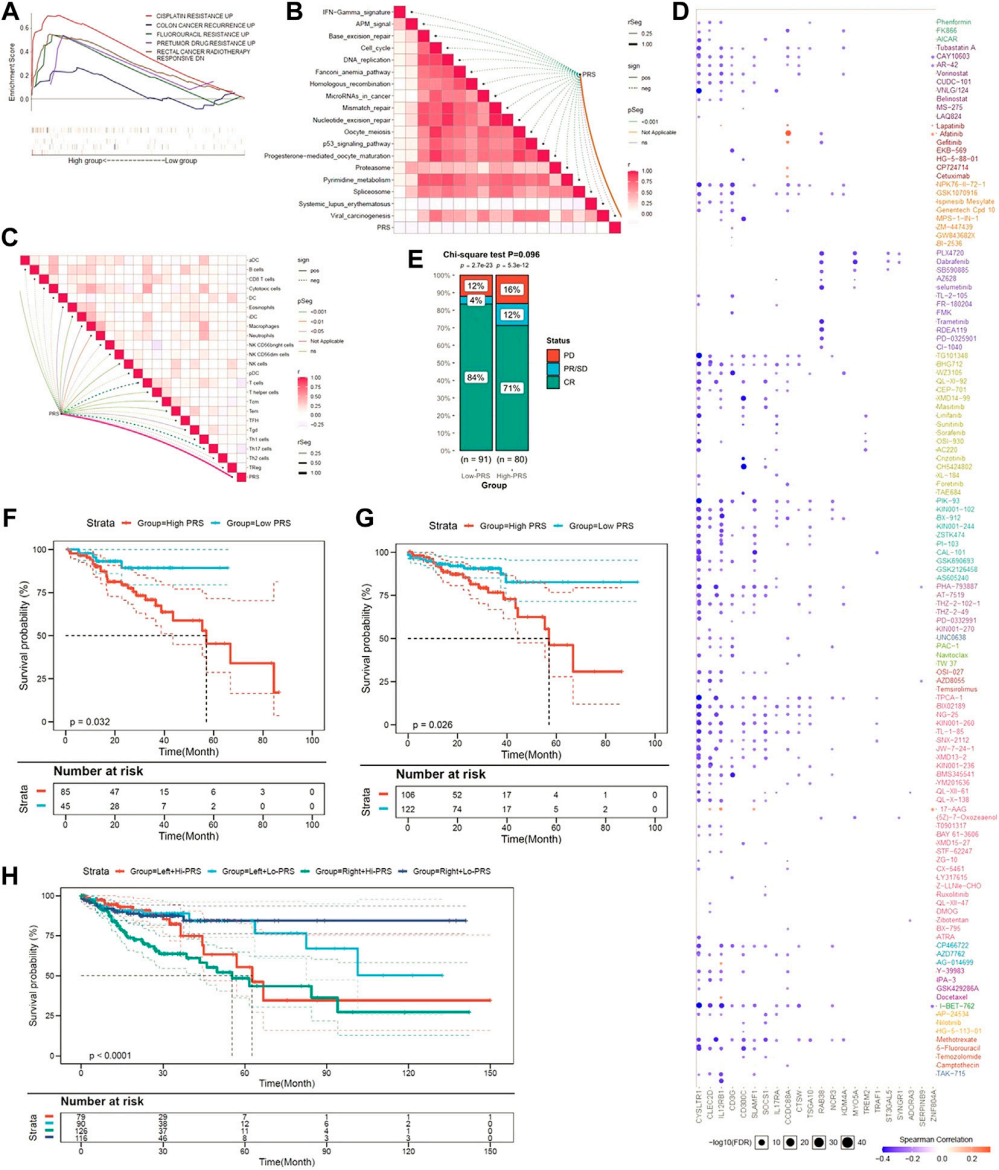
The pyroptotic-related gene signature is a promising marker of therapeutic resistance. **(A)** GSEA confirmed that the gene signature was associated with therapeutic resistance. **(B)** PRS is negatively associated with virous cancer therapeutic pathways. **(C)** PRS is related to immunosuppressive cells. **(D)** A landscape plot was conducted to depict the relationships between different molecules and the pyroptosis-related gene signature. **(E)** The ratio of worse outcomes after surgery is greatly elevated in higher PRS group. **(F–H)** Low-PRS is a prognostic marker of a more favorable outcome in different subgroups (6F: drugs; 6G: surgery; 6H: location of primary tumor).

Since limited evidence can be reached, we explored whether the pyroptosis-related genes signature contributed to disease recurrence and chemotherapy resistance. As shown in Figure 6A, GSEA suggested that higher PRS is not only significantly associated with resistance to chemotherapy drugs, including cisplatin and fluorouracil, but also with disease recurrence.

### PRS was associated with immune cells, rather than cancer therapeutic pathways

Further functional assays indicated that PRS was negatively associated with virous cancer therapeutic pathway (Figure 6B). In addition, we found PRS was positively related to immune cells, such as induced dendritic cells (iDC), macrophages, neutrophils, and natural killer (NK) cells. In contrast, PRS was negatively associated with Th1, Th2, Th17, and Treg cells (Figure 6C).

By using GSCALite, a landscape plot was generated to depict the relationships between the response to drugs and pyroptosis-related genes signature (Figure 6D). The results were presented as the bubble heatmap. Specifically, CCDC88A was associated with multi-drug resistance, while CYSLTR1, CLECC2D and CD3G contributed to drug sensitivity.

Medical information from TCGA were used to validate the prediction. As Figure 6E shown, although statistical difference was not reached, the disease control rate (DCR) was much higher in patients with low-PRS (88 vs. 83%, *p* = 0.092). Moreover, low-PRS is a prognostic marker of more favorable outcome among CC patients who received anti-cancer drugs (*p* = 0.032) (Figure 6F) or surgery (*p* = 0.026) (Figure 6G). When patients were stratified by the location of tumor, low-PRS group still had a survival advantage over high-PRS group (*p* < 0.0001) (Figure 6H).

### Combination of the pyroptosis signature and clinicopathological features improves risk stratification and survival prediction

**FIGURE 7:**
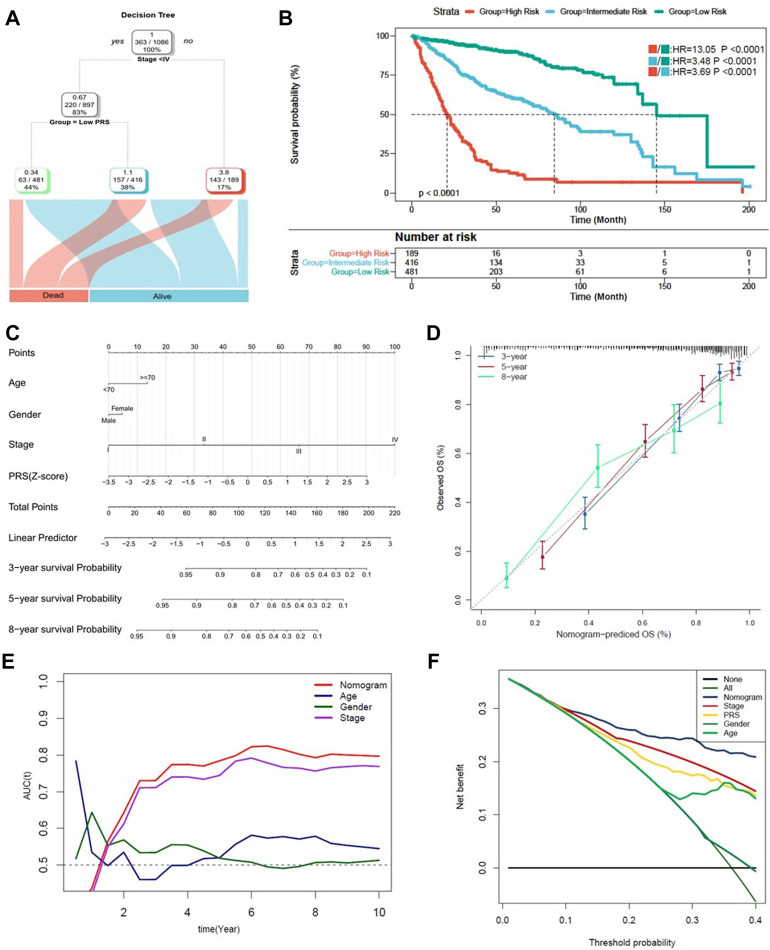
Combination of the pyroptosis signature and clinicopathological features improves risk stratification and survival prediction. **(A)** A decision tree was constructed to improve risk stratification. **(B)** Performance of the decision tree. **(C)** A nomogram was constructed to quantify risk assessment for individual patients. **(D)** Calibration analysis indicated a high accuracy of survival prediction. **(E,F)** tROC analysis and decision curve analysis (DCA) demonstrated that the nomogram was the most stable and powerful predictor for OS among all the clinical variables.

Finally, a decision tree improving risk stratification for OS was constructed, as 1,096 patients with four parameters available, age (>70 or ⩽70), sex (male or female), TNM stage (<IV or ≥ IV) and PRS (low or high) were included. The results indicated that only TNM stage and PRS remained, as three different risk subgroups were identified (Figure 7A). Furthermore, in the node of stage < IV, PRS took the place of TNM stage. Kaplan-Meier analysis indicated that statistical difference of OS was reached among the three subgroups (*p* < 0.0001) (Figure 7B).

A nomogram with PRS combined with other clinicopathological features was subsequently developed, aiming to quantify the risk assessment and survival probability for individual CC patients (Figure 7C). In the calibration analysis, all the prediction lines closed to the ideal performance (45-degree dotted line) (Figure 7D), validating the accuracy of the nomogram. As shown in Figure 7E, tROC analysis indicated that nomogram was the most powerful predictive factor. The clinical benefit of the nomogram was also demonstrated in decision curve analysis (DCA) (Figure 7F).

## Discussion

In current study, we identified pyroptosis was an independently risk factor for CC patients. Then combined methods were used to construct a pyroptosis-related genes prognostic model. The prognostic value of PRS, derived from the gene signature, was validated in independent cohorts. Further analysis suggested that PRS could be served as an independent risk factor to identify patient population with high-risk. Functional analyses indicated that PRS was negatively associated with virous pathways, but related to immune cells.

Pyroptosis, an inflammatory type of regulated cell death, is characterized by cell swelling, lysis, and the release of many proinflammatory factors, such as IL-1β and IL-18 (Tang et al., 2020). In the past decade, the relationship between pyroptosis and cancer development have attracted widespread attention. Existing evidence suggested that pyroptosis may impact the proliferation, invasion, and metastasis of tumor, making it a promising therapeutic target (Shi et al., 2015; Wang et al., 2018a; Yu et al., 2021). For example, by comparing samples from patients with esophageal cancer, Wang F et al. identified that pyroptosis promoted the progression of esophageal cancer, as the activation levels of pyroptosis-associated factorss, caspase-1, IL-1β and IL-18, were elevated in cancer tissues (Wang et al., 2018a). This finding has been conquered in cervical cancer, since cervical cancer cells released more IL-18 and IL-1β than normal cervical epithelial cells (Yu et al., 2021). Also, some individual pyroptosis genes have been studied, such as NOD2 in colorectal cancer (Couturier-Maillard et al., 2013; Branquinho et al., 2016), gasdermin B (GSDMB) in digestive system (Zhou et al., 2020), and Gasdermin D (GSDMD) in gastric cancer (Wang et al., 2018b). But how pyroptosis-related genes interact and whether they are related to the survival of patients with cancer remained little known. In addition, the influence of gene signatures may vary in different cancer types. Inconsistent with previous studies, lower expression of pyroptosis-associated factors was identified in HCC (Chen et al., 2018). Thus, it is worthy to establish a pyroptosis-related risk score for patients with CC.

Here, we developed a pyroptosis-related gene panel to predict the prognosis of CC patients. Previously, a constructed study was performed by Zhuang Z et al. to explore the prognostic value of pyroptosis-related genes in patients with CC (Zhuang et al., 2021). Similar results were conquered in our study. Notably, little different from Zhuang Z’s study, more microarray datasets were applied in our studies. Dataset GSE14333 was used as train set, and another five datasets from GEO were integrated into a new validation cohort, as well as dataset from TCGA. And we subsequently constructed a decision tree to improve risk stratification for survival, while a nomogram was built to quantify risk assessment and survival probability. In addition, more comprehensive gene panel was used in our study, compared with a five pyroptosis-related gene signature for HCC and a seven-gene signature for OC (Ye et al., 2021; Zheng et al., 2021).

Among genes in our panel, few of them showed evident correlations with cancer or pyroptosis in previous studies. The protective value of ACOT11, ST3GAL5, and SERPINB9 has been validated in renal cell carcinoma, bladder cancer and colorectal cancer (Vycital et al., 2019; Ouyang et al., 2020L; Xu et al., 2020), while MYO5A, RAB38, and CYSLTR1 were involved in cancer progression (Ohd et al., 2003; Lan et al., 2010; Hsieh et al., 2019; Li et al., 2019). Regarded as an endogenous inhibitor of Granzyme B, higher expression of SERPINB9 in patients with colorectal cancer was associated with superior OS (Vycital et al., 2019). Meanwhile, Xu CL et al. used different microarray dataset to measure the expressions of ACOTs in paired normal and clear cell renal cell cancer (ccRCC) tissues. The results confirmed the high diagnostic value of ACOT11 for this patient population, since its expression was significantly downregulated in ccRCC samples. Further survival analysis indicated ccRCC patients with low expression of ACOT11 seems had better OS than its competitor, although the *p* value did not reach statistical significance (Xu et al., 2020). On the other hand, MYO5A has been found to partake in the metastasis of cancer, as it was positively correlated with the expression of Snail, which could trigger the epithelial-mesenchymal transition (Lan et al., 2010). As a member of RAB family, the cancer promoting effects of RAB38 has been verified in pancreatic cancer, since RAB38 was correlated with progression in patients with pancreatic adenocarcinoma. And downregulation of RAB38 could suppressed the proliferation and migration of pancreatic cancer cells (Li et al., 2019). As for SOCS1, a biomarker with the largest protective coefficient in our study, was widely recognized as a tumor suppressor. However, its role in CRC remains controversial. Hanada T et al. found, through the IFN gamma/STAT1 pathways, SOCS1 could prevents chronic inflammation-mediated carcinogenesis (Hanada et al., 2006). Inconsistent with this finding, another study suggested, that SOCS1 may work as an oncogene in CRC (Tobelaim et al., 2015). Therefore, the biological functions of pyroptosis-related genes require further investigation in CC.

After the selection of pyroptosis-related genes signature, the PRS was constructed. Our findings were in line with published studies. Patients with lower PRS had favorable survival, compared with those with higher PRS. Potential reasons contributed to this finding, as higher PRS was significantly associated with drugs resistance, as well as disease recurrence. Previous studies have been powered to investigate the involvement of pyroptosis in cancer treatment (Westbom et al., 2015; Wang et al., 2020b). For example, Wang X et al. found, the treatment of Taxol triggered pyroptotic death in nasopharyngeal cancer cell line, which was mediated by Caspase-1 and GSDMD (Wang et al., 2020b). Meanwhile, based on a series of experiments conducted by Westbom C et al., the results suggested that doxorubicin and cisplatin could activate Caspase-1 and pyroptosis, inducing the death of cancer cells (Westbom et al., 2015). As limited evidence implicated the correlation between pyroptosis and disease recurrence, our findings need to be confirmed.

Over time, there has been an increasing interest to explore the impact of pyroptosis on tumor immune microenvironment. Study conducted by Wang Q et al suggested, tumor-suppressed immune cells are recruited by tumor cells undergoing pyroptosis (Wang et al., 2020a), which was in line with the study performed by Zhang Z et al. (Zhang et al., 2020). Meanwhile, Wang et al. found, with the concomitant induction of pyroptosis, immunotherapy could efficiently kill a specific group of tumor cells, natural resistant to immunotherapy (Wang et al., 2020a). Previous studies confirmed the predictive value of PRS in immune response. After stratified by PRS, less immune cells infiltrated in patients with higher scores, or patients with low-score usually showed better response to immunotherapy. Interestingly, Ye Y et al. found, higher proportion of Treg was found in the low-risk group (Ye et al., 2021). As Treg was redeemed as immunosuppressive and associated with poor outcomes, possible reason for this discrepancy is, the regulation of the inflammatory reaction caused by pyroptosis requires Treg. In our study, functional assays indicated PRS is closely related to immunosuppressive cells, including Treg. Notably, Saito T identified two main subtypes of Treg in colon cancers, which had opposite roles in the regulation of the tumor microenvironment (Saito et al., 2016). Therefore, it is worthy to further identify the specific subtype of Treg correlated to PRS.

Currently, our study is not without limitations. This study is limited by its retrospective nature. As little evidence could be reached, our findings should be conquered by further studies. And Basic experiment is necessary to verity the molecular mechanisms.

## Conclusion

In summary, we established a pyroptosis-related genes signature to discriminate CC patients with different risk and predict the survival outcomes of this patient population. Further decision tree and nomogram confirmed the predictive value of pyroptosis-related genes signature. Our model could be a useful tool to select high-risk CC patients and facilitate individual management of CC.

## Data Availability

The datasets presented in this study can be found in online repositories. The names of the repository/repositories and accession number(s) can be found in the article/Supplementary Material.
