# Supplementary material for: Identification pyroptosis-related gene signature to predict prognosis and associated regulation axis in colon cancer
Source: Front Pharmacol. 2022 Sep 27;13:1004425. doi: 10.3389/fphar.2022.1004425 (PMC9559861; doi:10.3389/fphar.2022.1004425)
Supplement: Supplementary file 1 [file DataSheet1.docx]

| Genes | Full name |
| --- | --- |
| AIM2 | Absent in melanoma 2 |
| CASP1 | cysteine-aspartic acid protease-1 |
| CASP3 | cysteine-aspartic acid protease-3 |
| CASP4 | cysteine-aspartic acid protease-4 |
| CASP5 | cysteine-aspartic acid protease-5 |
| CASP6 | cysteine-aspartic acid protease-6 |
| CASP8 | cysteine-aspartic acid protease-8 |
| CASP9 | cysteine-aspartic acid protease-9 |
| ELANE | elastase, neutrophil expressed |
| GPX4 | glutathione peroxidase 4 |
| GSDMA | gasdermin A |
| GSDMB | gasdermin B |
| GSDMC | gasdermin C |
| GSDMD | gasdermin D |
| GSDME | gasdermin E |
| IL18 | interleukin 18 |
| IL1B | interleukin 1 beta |
| IL6 | interleukin 6 |
| NLRC4 | NLR family CARD domain containing 4 |
| NLRP1 | NLR family pyrin domain containing 1 |
| NLRP2 | NLR family pyrin domain containing 2 |
| NLRP3 | NLR family pyrin domain containing 3 |
| NLRP6 | NLR family pyrin domain containing 6 |
| NLRP7 | NLR family pyrin domain containing 7 |
| NOD1 | nucleotide binding oligomerization domain containing 1 |
| NOD2 | nucleotide binding oligomerization domain containing 2 |
| PJVK | pejvakin/deafness, autosomal recessive 59 |
| PLCG1 | phospholipase C gamma 1 |
| PRKACA | protein kinase cAMP-activated catalytic subunit alpha |
| PYCARD | PYD and CARD domain containing |
| SCAF11 | SR-related CTD associated factor 11 |
| TIRAP | TIR domain containing adaptor protein |
| TNF | tumor necrosis factor |

Table S1 Pyroptosis-related genes

**Table S2(A) Baseline 14333**

|  | High-PRS | Low-PRS | p |
| --- | --- | --- | --- |
| N (%) | 105 | 121 |  |
| Gender |  |  | 1 |
| Female | 49 (46.7) | 57 (47.1) |  |
| Male | 56 (53.3) | 64 (52.9) |  |
| Age (mean (SD) | 64.54 (13.98) | 67.32 (12.02) | 0.109 |
| Stage (%) |  |  | <0.001 |
| Stage I | 9 (8.6) | 32 (26.4) |  |
| Stage II | 42 (40.0) | 52 (43.0) |  |
| Stage III | 54 (51.4) | 37 (30.6) |  |
| Stage IV | 0 | 0 |  |

**Table S2(B) Baseline 17536**

|  | High-PRS | Low-PRS | p |
| --- | --- | --- | --- |
| N (%) | 81 | 96 |  |
| Gender |  |  | 0.167 |
| Female | 32 (39.5) | 49 (51.0) |  |
| Male | 49 (60.5) | 47 (49.0) |  |
| Age (mean (SD) | 64.65 (12.50) | 66.18 (13.58) | 0.442 |
| Stage (%) |  |  | 0.003 |
| Stage I | 3 (3.7) | 21 (21.9) |  |
| Stage II | 25 (30.9) | 32 (33.3) |  |
| Stage III | 31 (38.3) | 26 (27.1) |  |
| Stage IV | 22 (27.2) | 17 (17.7) |  |

**Table S2(C) Baseline 41258**

|  | High-PRS | Low-PRS | p |
| --- | --- | --- | --- |
| N (%) | 141 | 110 |  |
| Gender |  |  | 1 |
| Female | 67 (47.5) | 52 (47.3) |  |
| Male | 74 (52.5) | 58 (52.7) |  |
| Age (mean (SD) | 63.75 (13.76) | 62.91 (14.94) | 0.643 |
| Stage (%) |  |  | <0.001 |
| Stage I | 17 (12.1) | 20 (18.2) |  |
| Stage II | 24 (17.0) | 35 (31.8) |  |
| Stage III | 33 (23.4) | 34 (30.9) |  |
| Stage IV | 67 (47.5) | 21 (19.1) |  |

**Table S2(D) Baseline TCGA**

|  | High-PRS | Low-PRS | p |
| --- | --- | --- | --- |
| N (%) | 216 | 216 |  |
| Gender |  |  | 0.289 |
| Female | 94 (43.5) | 106 (49.1) |  |
| Male | 122 (56.5) | 110 (50.9) |  |
| Age (mean (SD) | 65.12 (13.22) | 67.47 (12.34) | 0.057 |
| Stage (%) |  |  | <0.001 |
| Stage I | 33 (15.3) | 43 (19.9) |  |
| Stage II | 66 (30.6) | 102 (47.2) |  |
| Stage III | 79 (36.6) | 47 (21.8) |  |
| Stage IV | 38 (17.6) | 24 (11.1) |  |

**Table S2(E) Baseline Train cohort**

|  | High-PRS | Low-PRS | p |
| --- | --- | --- | --- |
| N (%) | 327 | 327 |  |
| Gender |  |  | 0.481 |
| Female | 148 (45.3) | 158 (48.3) |  |
| Male | 179 (54.7) | 169 (51.7) |  |
| Age (mean (SD) | 64.23 (13.50) | 65.50 (13.61) | 0.231 |
| Stage (%) |  |  | <0.001 |
| Stage I | 29 (8.9) | 73 (22.3) |  |
| Stage II | 91 (27.8) | 119 (36.4) |  |
| Stage III | 118 (36.1) | 97 (29.7) |  |
| Stage IV | 89 (27.2) | 38 (11.6) |  |
